# Risk of Mental Disorders in Children and Adolescents With Atopic Dermatitis: A Systematic Review and Meta-Analysis

**DOI:** 10.3389/fpsyg.2019.01773

**Published:** 2019-08-06

**Authors:** Qian-Wen Xie, Xiaolu Dai, Xinfeng Tang, Celia H. Y. Chan, Cecilia L. W. Chan

**Affiliations:** ^1^Department of Social Work and Social Administration, The University of Hong Kong, Pokfulam, Hong Kong; ^2^Department of Social Work and Social Administration, Changsha Social Work College, Changsha, China

**Keywords:** pediatric atopic dermatitis, children and adolescents, mental disorders, eczema, integrative medicine

## Abstract

Assessing the psychological effects on children and adolescents of suffering atopic dermatitis (AD) is essential, when planning successful management. This study aimed to systematically review the literature regarding risk of mental disorders in children and adolescents with, or without, AD; and to explore confounders. We identified potentially relevant studies from EMBASE, MEDLINE, PsycINFO, ERIC, the British Nursing Index, the Family and Society Studies Worldwide, the Social Work Abstracts, and the Sociological Abstracts from inception to Sep 30, 2018. Investigators independently screened titles and abstracts, and then full-texts. Investigators independently extracted data from included studies. Meta-analyses using random-effects models were performed, reporting odds ratios (ORs; 95% CIs). Thirty-seven studies (*n* = 2,068,911 children/ adolescents) were included. Meta-analysis of 35 studies found that children and adolescents with AD had significantly higher risk of total mental disorders than those without AD (OR = 1.652; 95% CI, 1.463–1.864). There was no significant difference in risks for ADHD (OR = 1.563; 95% CI, 1.382–1.769); sleep disorders (OR = 2.100; 95% CI, 1.322–3.336); anxiety (OR = 1.339; 95% CI, 1.062–1.687); depression (OR = 1.402 95% CI, 1.256–1.565); conduct disorder (OR = 1.494 95% CI, 1.230–1.815); or ASD (OR = 2.574; 95% CI, 1.469–4.510; *Q*_b_ = 8.344, *p* = 0.138). Race/ethnicity of child, target of comparison, type of studies, representativeness of the sample, measures of AD and mental disorders were significant moderators for total mental disorders. Integrated, holistic, multidisciplinary management of pediatric AD is significantly important, which emphasizes the well-being of the whole person.

## Introduction

Atopic dermatitis (AD) is the most common skin diseases in the pediatric population that affects about 15–30% children and adolescents worldwide (Archer, 2013). AD mostly occurs in early childhood, which is a critical period of life for physical and psychological development (Bronkhorst et al., 2016). The complex pathogenesis of AD may underpin the lack of effective medical treatment for many cases, with one-third of affected children continuing to experience symptoms in adolescence (Mitchell et al., 2015).

The link between pediatric AD and mental disorders, especially attention-deficit/hyperactivity disorder (ADHD), has attracted increasing attention in the past decade, due to parallel rises in their global prevalence (Nygaard et al., 2016). Also, mental disorders and skin diseases are in the top 20 conditions with the highest personal healthcare costs for children and adolescents in some developed countries such as the United States (Bui et al., 2017). It is becoming more apparent that healthcare professionals should understand the psychological effects on children of suffering AD in order to treat it successfully (Dertlioglu et al., 2012).

However, the overall trends of mental disorders in children with AD are not clear. Important knowledge gaps exist in the area of research. There are inconsistent findings regarding the risk of mental disorders in children with AD (Cheng et al., 2015; Lee et al., 2016). Most previous research has focused on associations between pediatric AD and one mental disorder only generally ADHD (Horev et al., 2017; Schans et al., 2017). A recent systematic review found the positive association between pediatric AD and depression (Rønnstad et al., 2018). However, it did not assess the risk of other important types of mental disorders in children with eczema such as anxiety, autism spectrum disorder (ASD), and conduct disorder, etc. Some relevant studies may be not included in this review since it only searched three medical databases. Moreover, no study to date has compared the risks of different types of mental disorders in children with AD. Additionally, previous research has suggested that AD prevalence is associated with demographic factors such as age, gender (Carson, 2013), race/ethnicity (Silverberg and Paller, 2015); socioeconomic factors such as family socioeconomic status (SES) (Mercer et al., 2004), and country SES (Stewart et al., 2001). Yet, no study has attempted to explore the impact of these potential confounders on the association between pediatric AD and mental disorders. Understanding different circumstances and contexts is essential when planning holistic health care for children suffering AD.

Therefore, this paper describes the findings of a systematic literature review and meta-analysis. Its objectives were to: (1) systematically review the available evidence and assess whether children and adolescents with AD have higher risks of mental disorders than children who do not suffer from AD; (2) determine risks of specific mental disorders for children suffering AD; and (3) analyze the effect of potential confounders by seeking differences in effect sizes related to participant and study characteristics.

## Materials and Methods

### Protocol and Registration

The study followed the systematic review protocol PROSPERO 2018: CRD42018087957 (Xie and Tang, 2018). Findings were reported using the Preferred Reporting Items for Systematic Reviews and Meta-Analyses diagnostic test accuracy (PRISMA-DTA) guideline (McInnes et al., 2018).

### Eligibility Criteria

English-language published, peer-reviewed scientific articles reporting primary experimental or observational studies were potentially eligible for inclusion. Studies should: (1) target children and adolescents (≤18 years old); (2) assess mental disorders categorized by the Diagnostic and Statistical Manual of Mental Disorders Fifth Edition (DSM-5; American Psychiatric Association, 2013) or outcome domains identified as “mental health problems” or “psychiatric disorders,” etc.; (3) compare the prevalence and/or incidence of mental disorders between children or adolescents with AD, and those without AD; and (4) apply quantitative research methods and report effect sizes, or data that could be extracted to compute effect sizes. Excluded were conference abstracts, secondary evidence or case reports.

### Information Sources

A systematic search of eight electronic databases was undertaken in September 2018, including EMBASE (from 1974), MEDLINE (from 1946), PsycINFO (from 1806), ERIC (from 1966), Social Work Abstracts (from 1968), Family and Society Studies Worldwide (from 1970), British Nursing Index (from 1994), and Sociological Abstracts (from 1952). The authors believed that these databases were most likely to contain papers appropriate to this review.

### Search

Three groups of search terms were used in combination and modified according to the requirements of the electronic databases: (1) AD (eczema OR atopic dermatitis OR atopic eczema OR neurodermatitis); (2) children (child^*^ OR boys OR girls OR juvenil^*^ OR minors OR adolesc^*^ OR preadolesc^*^ OR pre-adolesc^*^ OR pre-school OR preschool OR pediatric^*^ OR pediatric^*^ OR pubescen^*^ OR puberty OR school^*^ OR campus OR teen^*^ OR young OR youth^*^); and (3) mental disorders (psychiatri^*^ OR psycho^*^ OR mental OR depress^*^ OR anxiet^*^ OR disorder^*^ OR therap^*^ OR counsel^*^). Search dates were from database inception to Dec 6, 2017. A final prepublication search of these databases was undertaken from Oct 1, 2017 to Sep 30, 2018. The reference lists of previous systematic reviews were hand-searched (Chida et al., 2008; Schmitt et al., 2010b; Schans et al., 2017; Rønnstad et al., 2018). All records were incorporated into this study.

### Study Selection

All records were exported into EndNote software (EndNote, RRID:SCR_014001). Two investigators (Q-WX and XT) independently screened titles and abstracts for eligibility, and two investigators (Q-WX and XD) independently assessed full texts of potentially relevant studies. Differences were resolved by discussing with a third investigator.

### Data Collection Process

Information on participant and study characteristics was independently extracted by two investigators (Q-WX and XD) by using a pre-piloted, standardized coding scheme (Table 1), demonstrating a high level of inter-rater agreement (mean Cohen’s Kappa = 0.90).

**TABLE 1:** Coding scheme.

**No.**	**Variable**	**Definitions and Conditions**
A	Sample size	Numbers of participants in AD group/Non-AD group
B	Age of child (mean)	Mean age (years) of participated children in total sample
C	Age of child (range)	Range of age of participated children in total sample
D	Sex of child	Percentage of females in total sample
E	Race/ethnicity of child	Percentage of racial or ethnic minorities in total sample
F	Asthma	Percentage of children who were identified as having asthma by the included study in AD group/Non-AD group
G	Home SES	Percentage of families which were defined as low SES by the included study in total sample
H	Location	Country or territory in which the study was conducted
I	Type of study	1 = cohort, 2 = case-control, 3 = cross sectional
J	Representativeness of the sample	1 = convenience sample or clinical sample, 2 = random sample or community sample
K	Target of comparison	Nature of the Non-AD group (1 = healthy control, 2 = general population, 3 = people with other conditions)
L	Equivalence	Equivalence of AD group and Non-AD group (1 = yes, 2 = no)
M	Assessment of AD	Method employed to identify AD of participants (1 = diagnosed by a doctor or professional, 2 = self/parental report of doctoral diagnosis, 3 = questionnaire)
N	Assessment of quality	Total score calculated according to the Newcastle-Ottawa Quality Assessment Forms
O	Outcome domains	Type of mental disorders of participated children identified by the included study
P	Measurement of outcome	Name of scales or other methods used to measure the mental disorders of participated children
Q	Rater of outcomes	People who rate the mental disorders of participated children (1 = child/adolescent, 2 = parent/caregiver, 3 = clinician)
R	Original metric	Original data used to calculate effect sizes (1 = OR, 2 = mean and SD, 3 = rates or events)

*Data of follow-up tests in cohort study; AD, Atopic dermatitis; OR, odds ratio; SD, standard deviation; SES, socioeconomic status.*

### Risk of Bias, and Applicability

Two investigators (Q-WX and XD) independently assessed the methodological quality of each study using the Newcastle-Ottawa Scale (NOS) for case-control and cohort studies (Wells et al., 2012), and cross-sectional studies (Herzog et al., 2013). The NOS assess three domains of quality for each included study, including the selection of sample, the section of comparability, and exposure or outcome. The highest score was 9 for a study when it satisfied all criteria.

### Synthesis of Results and Meta-Analysis

All calculations were performed using Comprehensive Meta-Analysis (CMA, RRID:SCR_012779) software and applying the random-effects models given the heterogeneity of the included studies (Lipsey and Wilson, 2001). A meta-analysis was performed to calculate an overall effect size of all studies combined, to illustrate the risk of *Total Mental Disorders* in children with AD, compared to those without AD. To do this, effect size ORs were first computed for each study. When one type of mental disorder was measured by multiple tests, findings were combined by averaging the effect sizes within that study. When a study contained more than one outcome domain, effect sizes of different outcome domains were aggregated by averaging means to generate a combined effect size. This avoided the risk of including more than one effect size per construct per sample (Lipsey and Wilson, 2001; Borenstein et al., 2009). Independent meta-analyses were performed, and subtotal effect sizes of relevant studies were combined to illustrate the risks of specific types of mental disorders (e.g., ADHD, sleep disorders, anxiety, depression, etc.) in children with AD, compared to those without AD. Crude odds ratios (ORs) were used to compare the relative likelihood of the occurrence of mental disorders, given exposure to AD. Precision of effect sizes was reported by 95% Confidence Intervals (CIs). A combined effect size was considered significant if the CI did not include 1 and the *p*-value was significant in the *Z*-test.

### Additional Analyses

#### Assessment of Heterogeneity

Heterogeneity across studies was computed using the Q statistic and quantified by the I-squared (*I*^2^) value (Borenstein et al., 2009).

#### Sensitivity Analyses

We performed sensitivity analyses by removing studies one-by-one to estimate the strength of association between pediatric AD and mental disorders.

#### Assessment of Publication Bias

Omitting unpublished studies from this meta-analysis could bias the estimates of risk of mental disorders in children with AD, because studies with significant findings might have more opportunities to be published in peer-reviewed journals than studies with Non-significant findings. Possible publication bias was tested using funnel plot asymmetry (Borenstein et al., 2009) and quantified by the Egger’s test (Sterne et al., 2001). The unbiased effect size was calculated using the trim and fill approach. The Rosenthal’s fail-safe number was applied to indicate the number of potentially missing studies with Non-significant results, that would have to be included in the meta-analysis before the *p*-value became Non-significant.

#### Subgroup Analysis

Each type of mental disorder was treated independently. Each outcome domain was also treated as an independent correlate, by comparing effect sizes for different types of mental disorders in children with AD, compared to those without AD.

#### Meta-Regression Analysis and Moderator Analysis

By using mixed effects models, the effect of potential confounders (moderators) were explored to explain the variability in effect sizes of *Total Mental Disorder* if the assumption of homogeneity between studies was rejected. Moderator analysis was conducted for categorical variables by comparing effect sizes for studies grouped by study classification, on the potential moderator.

## Results

### Study Selection

**FIGURE 1:**
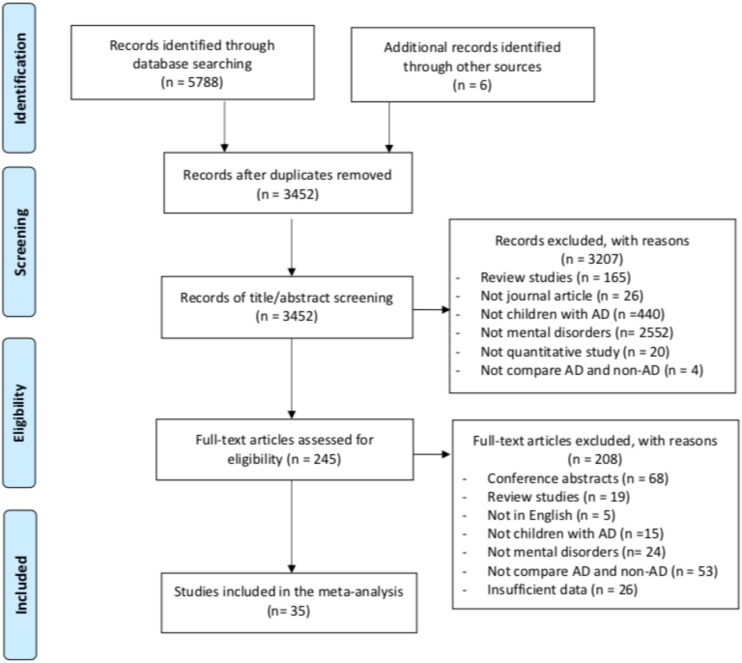
Selection of articles for inclusion.

The electronic database search identified 5,788 records. Hand-searching reference lists of earlier systematic reviews yielded six additional relevant studies. After de-duplication, 3,452 studies remained for title and/or abstract screening, following which 3,207 studies were excluded as not being relevant. Further excluded were 200 studies after the full-texts of 245 potentially eligible studies had been reviewed. Thirty-seven studies were selected after full-text screening. Two papers only provided adjusted hazard ratios (HRs) from which effect sizes could not be determined (Cheng et al., 2015; Riis et al., 2016). Sensitivity analyses (conducted by removing papers one at a time) indicated no significant change of the overall results. Therefore, thirty-five studies were included in the current meta-analysis. Figure 1 outlines the process of study selection.

### Study Characteristics

Table 2 presents characteristics of participants and studies. Outcomes and measures are reported in Table 3.

**TABLE 2:** Characteristics of participants and studies.

**Study name**	**A**	**B**	**C**	**D**	**E**	**F**	**G**	**H**	**I**	**J**	**K**	**L**	**M**	**N**
Absolon et al., 1997	30/30	9.3	5–15	51.7	53.5	47.0/10.0	21.5	United Kingdom^*^	2	1	3	1	1	6
Augustin et al., 2015	30354/263827	NR	0–18	48.8	NR	NR	NR	Germany^*^	3	2	3	2	1	6
Beyreiss et al., 1988	81/81	NR	5–12	NR	NR	NR	NR	NR	2	NR	NR	1	NR	3
Brew et al., 2018	3152/11045	9.0	9	49.6	NR	NR	NR	Sweden^*^	1	2	2	2	3	6
Buske-Kirschbaum et al., 1997	15/15	11.9	9–14	53.3	NR	NR	NR	Germany^*^	2	1	1	1	1	7
Camfferman et al., 2010	77/30	9.9	6–16	50.2	NR	NR	NR	Australia^*^	2	1	1	1	1	6
Catal et al., 2016	80/74	2.1	3–	48.6	NR	NR	28.6	Turkey	2	1	2	1	1	3
Chang et al., 2013	84/473	4.8	3–7	48.7	NR	NR	40.7	Korea^*^	3	2	2	2	3	7
Chun et al., 2015	325/2867	15.1	10–18	43.4	100	NR	16.9	Korea^*^	3	2	2	2	2	8
Covaciu et al., 2013	508/2648	8.0	8	49.7	NR	NR	NR	Sweden^*^	1	2	2	2	1	7
Genuneit et al., 2014	200/570	11.0	11	50.1	NR	NR	NR	Germany^*^	1	2	2	2	1	8
Horev et al., 2017	840/900	10.2	2–18	49.2	NR	NR	43.5	Israel^*^	2	1	3	1	1	7
Johansson et al., 2017	1178/2428	NR	10–18	49.4	NR	NR	16.4	Sweden^*^	1	2	2	2	2	9
Khandaker et al., 2014	994/5121	12.9	13	53.2	2.0	NR	NR	United Kingdom^*^	1	2	2	1	2	7
Kuniyoshi et al., 2018	1641/8313	NR	7–14	50.0	100	NR	NR	Japan^*^	3	2	2	2	3	7
Lee et al., 2016	18473/18473	NR	3–13	46.2	100	38.3/17.1	NR	Taiwan^*^	2	2	3	1	1	9
Lee and Shin, 2017	4904/67531	NR	12–17	53.0	100	NR	10.9	Korea^*^	3	2	2	1	2	8
Liao et al., 2016	387262/387262	NR	6–10	47.4	100	NR	30.3	Taiwan^*^	1	2	3	1	1	9
Lien et al., 2010	1030/2320	NR	15–16	56.7	NR	NR	2.4	Norway^*^	3	1	2	2	3	7
Romanos et al., 2010	1952/11366	9.9	3–17	50.4	NR	12.6/3.9	25.6	Germany^*^	3	2	2	2	2	8
Sarkar et al., 2004	22/20	4.8	3–9	40.5	NR	NR	NR	India	2	1	1	1	1	6
Schmitt et al., 2009	1436/1436	12.6	6–17	59.9	NR	10.3/2.9	NR	Germany^*^	2	2	3	1	1	7
Schmitt et al., 2010a	780/2136	10.0	10	49.0	NR	12/5	8	Germany^*^	1	2	2	2	1	7
Schmitt et al., 2011	367/1162	10.0	10	48.5	NR	11/4	14	Germany^*^	1	2	2	1	2	8
Schmitt et al., 2018	42/47	9.9	6–12	36.5	NR	NR	11.4	Germany^*^	3	1	1	2	1	5
Shani-Adir et al., 2009	57/37	7.0	4–10	50.8	NR	NR	NR	Israel^*^	2	1	1	2	1	4
Shyu et al., 2012	10620/178093	NR	0–17	47.8	100	NR	NR	Taiwan^*^	1	2	3	2	1	9
Silverberg and Simpson, 2013	10333/69334	8.0	0–17	49.1	45.2	25.1/12.3	17.9	United States^*^	3	2	2	2	2	7
Slattery and Essex, 2011	48/197	13.0	13	34.7	11	NR	NR	United States^*^	1	2	3	2	3	7
Strom and Silverberg, 2016	33808/302955	NR	2–17	NR	NR	NR	NR	United States^*^	1	2	2	2	2	7
Afsar et al., 2010	36/36	11.6	9–16	41.7	NR	NR	NR	Turkey	2	1	2	1	1	5
Urrutia-Pereira et al., 2017	340/454	6.8	4–10	NR	100	NR	NR	#	2	1	1	1	1	7
Wang et al., 2017	95/434	2.0	1–2	48.9	NR	NR	27.3	China	3	1	3	1	1	7
Yaghmaie et al., 2013	10401/69095	NR	0–18	48.6	44.5	25.2/NR	18.1	United States^*^	3	2	2	2	2	7
Yang et al., 2018	411/2361	NR	3–6	46.6	100	NR	32	Taiwan^*^	3	2	2	2	2	7

**(A)**, sample size: numbers of participants in AD group/Non-AD group; **(B)**, age of child (mean): mean age (years) of participated children in total sample; **(C)**, age of child (range): range of age of participated children in total sample; **(D)**, sex of child: percentage of females in total sample; **(E)**, race/ethnicity of child: percentage of racial or ethnic minorities in total sample; **(F)**, asthma: Percentage of children who were identified as having asthma by the included study in AD group/Non-AD group; **(G)**, home socioeconomic status (SES): percentage of families which were defined as low SES by the included study in total sample; **(H)**, location: country or territory in which the study was conducted; **(I)**, type of study: 1 = cohort, 2 = case-control, 3 = cross sectional; **(J)**, representativeness of the sample: 1 = convenience sample or clinical sample, 2 = random sample or community sample; **(K)**, target of comparison: nature of the Non-AD group, 1 = healthy control, 2 = general population, 3 = people with other conditions; **(L)**, equivalence: equivalence of AD group and Non-AD group, 1 = yes, 2 = no; **(M)**, assessment of AD: method employed to identify AD of participants; 1, diagnosed by a doctor or professional; 2, self/parental report of doctoral diagnosis; 3, questionnaire; **(N)**, assessment of quality: total score calculated according to the Newcastle-Ottawa Quality Assessment Forms; NR, not report; **#**Nine countries in Latin America including Argentina, Brazil, Colombia, Cuba, Dominican Republic, Honduras, Mexico, Paraguay, and Uruguay; ^*^developed countries or territories defined as those possessing an Human Development Index [HDI] over 0.800 according to United Nations Development Programme [UNDP] (2018).

**TABLE 3:** Outcomes and measurements.

**Study name**	**Outcome domains**	**Measurement of outcome**	**Rater of outcomes**	**Original metric**
Absolon et al., 1997	Psychological disturbance (including sleep disturbance)	Rutter A2 scale	Clinician	Rates or events
Augustin et al., 2015	ADHD, depression	Diagnosed	Clinician	OR
Beyreiss et al., 1988	ADHD	Parents Rating Scale	Parent/caregiver	Rates or events
Brew et al., 2018)	Anxiety/depression	SCARED, SMFQ	Parent/caregiver	OR
Buske-Kirschbaum et al., 1997	Anxiety	Anxiety Inventory for Children	Child/adolescent	OR
Camfferman et al., 2010	ADHD, sleep disorder	SDSC, Conners Parent Rating Scale-Revised	Parent/caregiver	Mean and SD
Catal et al., 2016	Psychiatric disorders (including ADHD, anxiety, attachment disorder, conduct disorder, eating disorder, ODD, sleep disorders, and tic disorders)	ECI-4	Parent/caregiver	Rates or events
Chang et al., 2013	ADHD, affective disorder, anxiety, externalizing problem, internalizing problem, ODD, PDD, and sleep disorders	CBCL	Parent/caregiver	Mean and SD
Chun et al., 2015	Depression	Self-designed questionnaire	Parent/caregiver	Rates or events
Covaciu et al., 2013	Anxiety/depression	Subscale of EQ-5D	Parent/caregiver	Rates or events
Genuneit et al., 2014	ADHD	Diagnosed	Clinician	Rates or events
Horev et al., 2017	ADHD	Diagnosed	Clinician	OR
Johansson et al., 2017	ADHD	Diagnosed	Clinician	OR
Khandaker et al., 2014	Psychotic experiences	PLIKSi	Child/adolescent	OR
Kuniyoshi et al., 2018	Mental health problems (including ADHD, and conduct disorder)	SDQ	Parent/caregiver	OR
Lee et al., 2016	ADHD, ASD	Diagnosed	Clinician	Rates or events
Lee and Shin, 2017	Depression	Self-designed questionnaire	Child/adolescent	Rates or events
Liao et al., 2016	ADHD, ASD	Diagnosed	Clinician	Rates or events
Lien et al., 2010	Internalizing and externalizing mental health problems	HSCL-10, SDQ	Child/adolescent	OR
Romanos et al., 2010	ADHD	Diagnosed	Clinician	OR
Sarkar et al., 2004	Psychological disorders (including anxiety, conduct disorder, depression)	CPMS	Parent/caregiver	Mean and SD
Schmitt et al., 2009	ADHD, affective disorder, eating disorder, personality disorder	Diagnosed	Clinician	Rates or events
Schmitt et al., 2010a	Mental health problems (including ADHD, and conduct disorder)	SDQ	Parent/caregiver	OR
Schmitt et al., 2011	Mental health problems (including ADHD, and conduct disorder)	SDQ	Parent/caregiver	OR
Schmitt et al., 2018	ADHD, mental health problems (including anxiety/depression), and sleep disorders	Conners 3 Rating Scales, CBCL, CSHQ, SSR	Child/adolescent and parent/caregiver	Mean and SD
Shani-Adir et al., 2009	Sleep disorders	CSHQ	Parent/caregiver	OR
Shyu et al., 2012	ADHD	Diagnosed	Clinician	OR
Silverberg and Simpson, 2013	Sleep disorders	Self-designed questionnaire	Parent/caregiver	Rates or events
Slattery and Essex, 2011	Anxiety, depression	HBQ, OCHS	Child/adolescent and parent/caregiver	Mean and SD
Strom and Silverberg, 2016	Speech disorder	Diagnosed	Clinician	Rates or events
Afsar et al., 2010	Anxiety	STAI-C	Child/adolescent	Mean and SD
Urrutia-Pereira et al., 2017	Sleep disorders	CSHQ	Parent/caregiver	Mean and SD
Wang et al., 2017	Sleep disorders	BISQ	Parent/caregiver	OR
Yaghmaie et al., 2013	ADHD, anxiety, ASD, conduct disorder, depression	Diagnosed	Clinician	Rates or events
Yang et al., 2018	ADHD	Diagnosed	Clinician	OR

*ADHD, attention-deficit/hyperactivity disorder; ASD, autism spectrum disorder; BISQ, Brief Infant Sleep Questionnaire; CBCL, Child Behavior Checklist; CPMS, Childhood Psychopathology Measurement Schedule questionnaire; CSHQ, Children’s Sleep Habits Questionnaire; Diagnosed, diagnosed by a doctor or professional; ECI-4, Early Childhood Inventory-4; HBQ, Health and Behavior Questionnaire (parental assessed); HSCL-10, The ten-item version of Hopkins Symptoms Checklist; OCHS, The Ontario Child Health Study scale (self-assessed by child); ODD, oppositional defiant disorder; PDD, pervasive developmental disorder, PLIKSi, Psychosis-like Symptoms interview; SCARED, Child Anxiety Related Emotional Disorders; SDSC, Sleep Disturbance Scale for Children; SMFQ, Shorted Mood and Feelings; SSR, Sleep Self Report (self-assessed by child); STAI-C, State-Trait Anxiety Inventory for Children; OR, odds ratio; SD, standard deviation.*

#### Characteristics of Participants

Data from 1,935,147 children and adolescents (521,976 identified as having AD) were included in this systematic review. Study sample size ranged from *n* = 30 to *n* = 774,524. Participants’ mean age was 9.12 years (*SD* = 3.29 years, *k* = 23). Similar numbers of boys and girls were reported (Mean % of females = 48.5%, *k* = 32). Three-quarters of subjects were Non-Caucasian (*M* = 73.6%, *k* = 13), and 10 studies were conducted totally on Asian populations (*n* = 1,089,330). AD diagnosis of children was confirmed by a doctor or other healthcare professional in most studies (*k* = 19). More than 20% of children with AD were also reported as having asthma (*M* = 22.7%, *k* = 8; compared with 7.8% in the Non-AD group. More than 20% of participating families were from low SES circumstances (*M* = 21.5%, *k* = 17). The majority of participants (*k* = 29, *n* = 1,933,466) were recruited from developed countries or territories with high Human Development Index (HDI) scores (8.00 or more; United Nations Development Programme [UNDP], 2018).

#### Characteristics of Studies

Eleven studies were published in 2010 or earlier. All studies applied observational study designs including 11 cohort studies, 12 case control studies, and 12 cross-sectional studies. In terms of representativeness of sample, 22 studies used a random sample or a community sample. Nineteen studies compared the prevalence or incidence of mental disorders in children with AD to the general population, rather than healthy controls.

#### Outcomes and Measures

Thirteen types of mental disorders were reported in the papers included in this systematic review including: ADHD (*n* = 1,414,406), sleep disorders (*n* = 82,051), anxiety (*n* = 3,881), depression (*n* = 449,591), conduct disorder (*n* = 94,091), autism spectrum disorder (ASD; *n* = 890,966), affective disorder (*n* = 3,429), eating disorder (*n* = 3,026), oppositional defiant disorder (ODD; *n* = 711), attachments disorder (*n* = 154), pervasive developmental disorder (PDD; *n* = 557), personality disorders (*n* = 2,872), speech disorder (*n* = 336,763), and tic disorders (*n* = 154). Mental disorders of children were diagnosed by clinicians or reported by parents/caregivers or children/adolescents.

### Risk of Bias and Applicability

The NOS total scores per-study ranged from 3 to 9 (N column in Table 2). Twenty-five studies had high methodological quality (NOS score of 7 or higher), whilst 10 studies had moderate quality (NOS score 3–6). The component NOS scores are reported in Supplementary Appendix 1.

### Meta-Analysis

**FIGURE 2:**
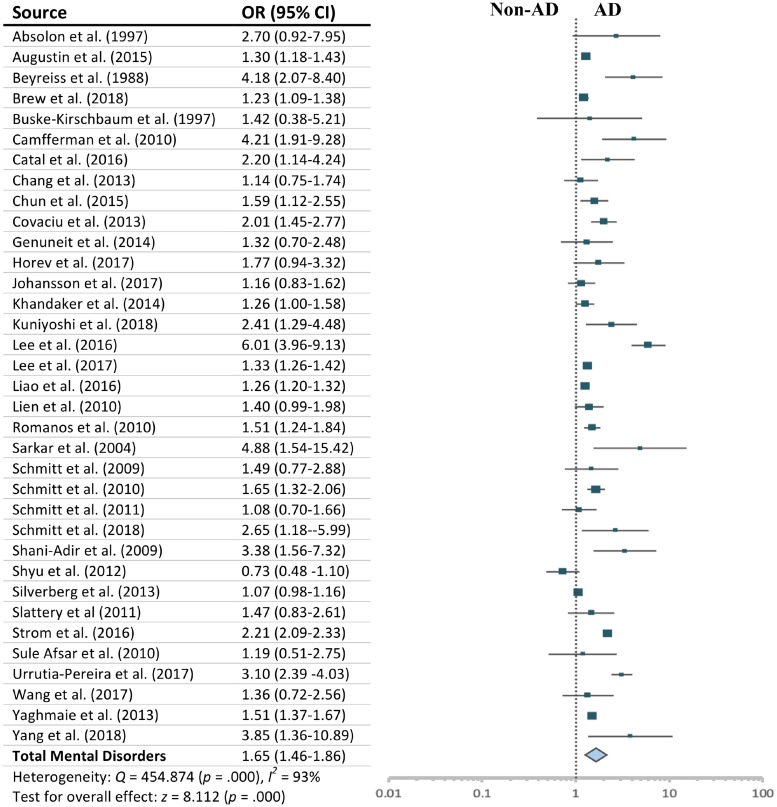
Effect sizes for total mental disorders.

Children and adolescents with AD had an overall statistically significant increased risk of total mental disorders compared with those without AD (OR = 1.652; 95% CI, 1.463–1.864; *Z* = 8.112, *p* < 0.001). The assumption of homogeneity was rejected (*Q* = 454.874, *p* = 0.000), as approximately 93% total variance among studies was due to heterogeneity (*I*^2^ = 92.53%). See Figure 2 for a summary of individual study effects and summary effects and the heterogeneity.

More specifically, compared with children without AD, suffering AD was significantly associated with higher risks of experiencing ADHD (OR = 1.563; 95% CI, 1.382–1.769; *Z* = 7.095, *p* < 0.001); sleep disorders (OR = 2.100; 95% CI, 1.322–3.336; *Z* = 3.144, *p* < 0.01); anxiety (OR = 1.339; 95% CI, 1.062–1.687; *Z* = 2.471, *p* < 0.05); depression (OR = 1.402; 95% CI, 1.256–1.565; *Z* = 6.012, *p* < 0.001); conduct disorder (OR = 1.494; 95% CI, 1.230–1.815; *Z* = 4.049, *p* < 0.001); and ASD (OR = 2.574; 95% CI, 1.469–4.510; *Z* = 3.305, *p* < 0.01) (see Supplementary Appendix 2). There was no statistically significant difference in risk of suffering any of these mental disorders (*Q*_b_ = 8.344, *p* = 0.138) in children with AD. Effect sizes for affective disorder, eating disorder, and ODD were not significant, and effect sizes for attachment disorder, PDD, personality disorder, speech disorder, and tic disorders are not presented, as there was only one study which reported on each of these outcomes.

### Publication Bias

Visual inspection of the funnel plot (see Supplementary Appendix 3) and the findings of the Egger test indicated no significant publication bias (*t* = 1.020, *p* = 0.315). The unbiased effect size (OR = 1.500; 95% CI, 1.329 to 1.694) was marginally smaller than the calculated effect size (OR = 1.652). The classic fail-safe number indicated that 4,591 studies with null findings needed to be added to negatively impact (overturn) the overall-effect *p*-value.

### Moderators of Risks of Mental Disorders in Children With AD

Total sample size (*Q*_b_ = 6.687, *p* = 0.083) and year of publication (*Q*_b_ = 2.336, *p* = 0.126) did not significantly contribute to between-group variance. Table 4 presents the analysis of potential categorical moderators.

**TABLE 4:** Moderator variables analysis.

**Moderators**	***k***	**Random Effect Size**	**Heterogeneity**		
		**OR (95% CI)**	***Q*_*w*_**	***p***	***I*^2^**
**Sample size**			***Q*_*b*_ = 6.687**	**0.083**	
Less than 100	6	2.419 (1.605, 3.647)	5.637	0.343	11.306
100–1000	8	2.046 (1.397, 2.997)	28.467	0.000	75.410
1001–10000	11	1.518 (1.308, 1.761)	16.572	0.084	39.656
More than 10000	10	1.477 (1.228, 1.777)	374.917	0.000	97.599
**Year of publication**			***Q*_*b*_ = 2.336**	**0.126**	
Earlier than or in 2010	11	1.944 (1.532–2.468)	21.936	0.015	54.413
Later than 2010	24	1.568 (1.365–1.802)	427.818	0.000	94.624
**Mean age**			***Q_*b*_* = 2.626**	**0.269**	
0–6 years	6	1.829 (1.118, 2.993)	29.474	0.000	83.036
7–11 years	11	1.641 (1.347, 1.999)	53.168	0.000	81.192
12–18 years	6	1.364 (1.151, 1.616)	1.430	0.921	0.000
**Sex of child**			***Q*_*b*_ = 0.099**	**0.753**	
Predominantly male (> 50%)	20	1.488 (1.316–1.681)	120.600	0.000	84.245
Predominantly female (> 50%)	11	1.536 (1.314–1.794)	19.937	0.030	49.843
**Race/ethnicity of child**			***Q_*b*_* = 4.963**	**0.026**	
Predominantly Caucasian (> 50%)	4	1.287 (1.017–1.630)	27.662	0.000	89.155
Predominantly minority (> 50%)	9	1.872 (1.487–2.355)	112.859	0.000	92.912
**Family SES**			***Q*_*b*_ = 0.418**	**0.518**	
More than 20% low SES families	8	1.458 (1.220, 1.743)	13.279	0.066	47.286
Less than 20% low SES families	9	1.356 (1.192, 1.543)	40.365	0.000	80181
**HDI in 2018**			***Q_*b*_* = 1.974**	**0.160**	
Developed countries or territories	29	1.559 (1.376, 1.768)	408.119	0.000	93.139
Developing countries or territories	5	2.190 (1.387–3.457)	10.518	0.033	61.971
**GNI per capital in 2017**			***Q*_*b*_ = 2.188**	**0.139**	
High income countries	25	1.528 (1.333, 1.752)	306.686	0.000	92.174
Middle or low income countries	5	2.190 (1.387, 3.457)	10.518	0.033	61.971
**Type of study**			***Q_*b*_* = 19.464**	**0.000**	
Cohort	11	1.362 (1.088–1.704)	262.520	0.000	96.191
Case-control	12	2.809 (2.081–3.790)	26.845	0.005	59.024
Cross-sectional	12	1.386 (1.242–1.547)	45.166	0.000	75.645
**Target of comparison**			***Q_*b*_* = 32.464**	**0.000**	
Health control	6	3.144 (2.525–3.195)	2.736	0.741	0.000
General population	19	1.477 (1.256–1.725)	286.948	0.000	93.727
People with other conditions	9	1.565 (1.239–1.977)	63.657	0.000	87.433
**Representativeness of the sample**			***Q_*b*_* = 6.359**	**0.012**	
Convenience sample/clinical sample	12	2.211 (1.671–2.925)	24.159	0.012	54.469
Random sample/community sample	22	1.483 (1.297–1.696)	392.730	0.000	94.653
**Equivalence of patients and control group**			***Q_*b*_* = 3.255**	**0.071**	
Yes	16	1.903 (1.548–2.287)	124.684	0.000	87.970
No	19	1.511 (1.274–1.792)	284.734	0.000	93.678
**Assessment of AD**			***Q_*b*_* = 8.089**	**0.018**	
Diagnosed by a doctor or professional	19	1.904 (1.562–2.322)	139.674	0.000	87.113
Self/parental report of doctoral diagnosis	10	1.439 (1.161–1.784)	262.994	0.000	96.578
Questionnaire	5	1.318 (1.118–1.553)	5.145	0.273	22.257
**Rater of mental disorders**			***Q*_*b*_ = 10.600**	**0.005**	
Child/adolescent	5	1.331 (1.257–1.409)	0.384	0.984	0.000
Parent/caregiver	15	1.849 (1.481–2.308)	114.477^∗∗∗^	0.000	87.770
Clinician	13	1.632 (1.321–2.018)	296.077^∗∗∗^	0.000	95.947
**Assessment of Quality**			***Q*_*b*_ = 2.507**	**0.113**	
High methodological quality	25	1.484 (1.445–1.525)	410.627	0.000	94.614
Moderate methodological quality	10	1.339 (1.245–1.441)	37.554	0.000	76.034

*k = number of studies; 95% CI = lower and upper limits if 95% confidence interval; *Qw/Qb* = test for homogeneity of effect sizes within (w) and between (b) groups.*

For demographic factors, race/ethnicity was a significant moderator (Predominantly minority race > Predominantly Caucasian; *Q*_b_ = 4.963, *p* = 0.026). However, mean age (*Q*_b_ = 2.626, *p* = 0.269) and gender (*Q*_b_ = 0.099, *p* = 0.753) did not significantly contribute to between-group variance.

For socioeconomic factors, family SES (*Q*_b_ = 0.418, *p* = 0.518), HDI (*Q*_b_ = 1.974, *p* = 0.160), and GNI per capital (*Q*_b_ = 2.188, *p* = 0.139) did not significantly contribute to between-group variance.

For methodological factors, the comparator group (healthy control group > people with other conditions > general population; *Q*_b_ = 32.464, *p* = 0.000), type of study (*Q*_b_ = 19.464, *p* = 0.000; case control studies > cohort studies > cross-sectional studies), and representativeness of the sample (convenience or clinical samples > randomly selected or community samples; *Q*_b_ = 6.359, *p* = 0.012) contributed significantly to between-group variance. Also, assessment of AD (diagnosed by a doctor or health professional > self/parental report of doctoral diagnosis > questionnaire; *Q*_b_ = 8.089, *p* = 0.018) and raters of mental disorders (parents or caregivers > clinicians > self-reported; *Q*_b_ = 10.600, *p* = 0.005) contributed significantly to between-group variance. However, equivalence of patients and control group (*Q*_b_ = 3.255, *p* = 0.071) and quality of studies (*Q*_b_ = 2.507, *p* = 0.113) were not statistically significant as moderators.

## Discussion

Skin as the largest organ of our body constitutes the boundaries between internal and external environments (Suárez et al., 2012). Besides biological symptoms, children with AD are more likely to encounter psychological challenges than their healthy peers. This meta-analysis of 35 studies (*n* = 1,935,147 children/adolescents) found that AD could result in higher risk of mental disorders among children and adolescents by providing conclusive evidence that children with AD were, on average, 65.2% more likely to develop mental disorders, compared with children without AD.

Previous studies have attempted to explore the mechanisms in which how AD impacts the psychological well-being of children. First, symptoms and characteristics of AD, such as severe and constant pruritus, chronic and relapsing nature, chronic inflammation, and high risk of atopic or allergic comorbidity may directly explain the elevated risk of mental disorders in children with AD (Silverberg and Simpson, 2013; Horev et al., 2017). Sleep disorders caused by pruritus may further strengthen the impact of AD on mental health of sufferers (Shyu et al., 2012). Pediatric AD may also indirectly induce mental disorders through dysfunctional social relationships (Bronkhorst et al., 2016; Chernyshov, 2016). In fact, recent studies have suggested that the relationships between suffering AD and mental disorders could be reciprocal (Chang et al., 2013; Becker-Haimes et al., 2017). Mental disorders may cause, induce, or exacerbate the physical symptoms of AD through decreased tolerance of pruritus, pain, and disfigurement; and lower adherence to medical treatment (Bronkhorst et al., 2016). Mental disorders can further cause adverse outcomes such as educational and career impairments, and increased risk for suicide in children with AD (Chun et al., 2015; Lee and Shin, 2017).

Topical therapies for treating physical symptoms are commonly the first line in the management of pediatric AD (Stein and Cifu, 2016). The high risk of mental disorders in children with AD, and the resultant negative consequences make it clear that successful management of pediatric AD requires a multipronged approach including psychological input, rather than just the medical treatments of physical symptoms (Ersser et al., 2014). Indeed, there has been growing interest in integrative medicine (IM) to manage pediatric AD in healthcare settings (Bodeker et al., 2017). This approach addresses healing of the whole person, including both physical, psychological, and social dimensions (Bell et al., 2002; McClafferty et al., 2017). There would also seem to be a strong indication that working with the psychological dimensions of children should be an integral and routine part of the management of AD (Bronkhorst et al., 2016). Mental health care adjunctive to medical treatments has great potential to help children with AD to manage itching and scratching, increase adherence to skin-directed care, develop coping competencies, accept their disease and themselves emotionally, and improve quality of life (Ersser et al., 2014). Addressing emotional distress of children with AD may be an important key to break the vicious cycle of AD and mental disorders in children.

The findings of this review suggest the need for greater inclusion of effective mental health care into holistic management of AD and the importance of multidisciplinary cooperation. On the one hand, health care professionals including dermatologists, allergists, pediatricians, and primary care physicians require a greater understanding of the risk of mental disorders in children with AD (Lee et al., 2016). When treating children with severe AD, screening procedures for identifying mental disorders should be emphasized in conjunction with medical treatment (Cheng et al., 2015). On another hand, mental health professionals including psychiatrists, psychologists, and social workers should give further attention to the symptoms of AD when treating mental disorders of children and adolescents, and also provide effective mental health care to children with AD. Additionally strategies such as listing and recommending mental health care in treatment or management guidelines of pediatric AD and increasing insurance coverage of mental health care for children in health care system should be considered.

Moreover, this study has significant implications for future practice and research. First, this study provided stronger evidence to point toward positive associations between pediatric AD and different types of mental disorders, such as ASD (OR = 2.574); sleep disorders (OR = 2.100); ADHD (OR = 1.563), conduct disorder (OR = 1.494), depression (OR = 1.402), and anxiety (OR = 1.339). The impacts of pediatric AD on different mental disorders were not shown to be significantly different. We suggest that psychological treatments need to target specific mental disorders of children with AD by using different techniques, rather than providing ubiquitous treatment, or just focusing on ADHD. Second, this study found that social-demographic factors including age, gender, and family/country SES did not moderate the impact of AD on mental disorders of sufferers. Mental health care should be developed for, and accessed by, young children and adolescents of both genders, from all family SES, and in both developed countries and developing countries. Third, more research focus is required on Non-Caucasian children since studies on children from minority races predominantly yielded larger effect size than those with predominantly Caucasian children. Fourth, this study found that studies with parents or caregivers as raters of mental disorders yielded larger effect size than those with clinicians or children themselves as raters. Providing daily care to a child with AD usually imposes a heavy psychological burden on parents due to the complex and long-term treatment management (Mitchell et al., 2015). The psychological stress of parents might exaggerate the degree of severity of children’s mental disorders in their reports. Only using parents or caregivers as the child’s proxy to report on the child’s health may induce bias. There is a need to involve the voices of children in future research and practice for moving closer to the best care appropriate for children.

### Limitations

First, a broad range of outcome measures was included to calculate the overall effect size of *Total Mental Disorders*. This resulted in large heterogeneity. We addressed this by conducting independent meta-analyses to assess the effect sizes related to specific mental disorders, and we also employed a more discriminatory random-effects model. Second, gray literature was not included in this meta-analysis. Due to our strict inclusion criteria, we were able to include only a limited number of studies. There were insufficient numbers of studies to support separate meta-analyses for some mental disorders, such as attachment disorders, personality disorders, and tic disorders. Third, the way we assessed for moderator impacts was exploratory, and the results should be interpreted with caution. We could not test for a number of important moderator variables, including medical factors (e.g., severity of AD, onset age and duration of AD, medical treatments received), immunologic factors (e.g., serum immunoglobulin [IgE] levels), genetic factors (e.g., family history of AD), and social factors (e.g., social relationships) because too few studies provided this information. More research on these important variables is needed for further investigating the mechanisms that how AD and mental disorders might be interrelated, which would be crucial for improved treatment of both disorders. Additionally, few studies provided information regarding the severity of mental disorders. Future research should explore the association between pediatric AD and different degrees of mental disorders, as not all children may require the same level of support.

## Conclusion

AD is a disease resulting in significant somatic suffering and psychological disturbance. This meta-analysis identified that children and adolescents with AD had higher risk of mental disorders compared to those without AD. This review highlighted the importance of integrated, holistic, multidisciplinary management of pediatric AD, which emphasizes the well-being of the whole person.

## Author Contributions

Q-WX conceptualized and designed the study, searched and screened the studies, extracted and analyzed the data, assessed quality of studies, wrote the initial draft of the manuscript, and significantly contributed to revision. XD screened the studies, extracted the data, assessed quality of the studies, and revised the manuscript. XT screened the studies. CLWC and CHYC critically reviewed and revised the manuscript, and significantly improved the manuscript quality. All authors approved the final version of the manuscript as submitted and agreed to be accountable for all aspects of the work.

## Conflict of Interest Statement

The authors declare that the research was conducted in the absence of any commercial or financial relationships that could be construed as a potential conflict of interest.
