# Supplementary material for: Risk of Mental Disorders in Children and Adolescents With Atopic Dermatitis: A Systematic Review and Meta-Analysis
Source: Front Psychol. 2019 Aug 6;10:1773. doi: 10.3389/fpsyg.2019.01773 (PMC6691144; doi:10.3389/fpsyg.2019.01773)

**Appendix 1. Assessment of Risk of Bias**

1. **Quality Assessment of Cohort Studies**

| **Study name** | **Selection** |  |  |  | **Comparability** | **Outcome** |  |  | **Total score** |
| --- | --- | --- | --- | --- | --- | --- | --- | --- | --- |
|  | **Exposed cohort** | **Non-exposed cohort** | **Ascertainment of exposure** | **Outcome of interest** | **Comparability of cohorts** | **Assessment** | **Length of follow-up** | **Adequacy of follow up** |  |
| Brew et al. (2018) | 1 | 1 | 0 | 1 | 2 | 0 | 1 | 0 | **6** |
| Covaciu et al. (2013) | 1 | 1 | 0 | 1 | 2 | 0 | 1 | 1 | **7** |
| Genuneit et al. (2014) | 1 | 1 | 1 | 1 | 2 | 0 | 1 | 1 | **8** |
| Johansson et al. (2017) | 1 | 1 | 1 | 1 | 2 | 1 | 1 | 1 | **9** |
| Khandaker et al. (2014) | 1 | 1 | 0 | 1 | 2 | 0 | 1 | 1 | **7** |
| Liao et al. (2016) | 1 | 1 | 1 | 1 | 2 | 1 | 1 | 1 | **9** |
| Schmitt et al. (2010) | 1 | 1 | 0 | 1 | 2 | 0 | 1 | 1 | **7** |
| Schmitt et al. (2011) | 1 | 1 | 1 | 1 | 2 | 0 | 1 | 1 | **8** |
| Shyu et al. (2012) | 1 | 1 | 1 | 1 | 2 | 1 | 1 | 1 | **9** |
| Slattery et al. (2011) | 1 | 1 | 0 | 1 | 2 | 0 | 1 | 1 | **7** |
| Strom et al. (2016) | 1 | 1 | 0 | 1 | 2 | 0 | 1 | 1 | **7** |

1. **Quality Assessment of Case-control Studies**

| **Study name** | **Selection** | | | | **Comparability** | **Exposure** | | | **Total score** |
| --- | --- | --- | --- | --- | --- | --- | --- | --- | --- |
|  | **Definition of cases** | **Representativeness of cases** | **Selection of controls** | **Definition of controls** | **Comparability of cases and controls** | **Ascertainment of exposure** | **Method of ascertainment** | **Non-Response rate** |  |
| Absolon et al. (1997) | 1 | 0 | 0 | 1 | 2 | 1 | 1 | 0 | **6** |
| Beyreiss et al. (1988) | 0 | 0 | 0 | 0 | 2 | 0 | 1 | 0 | **3** |
| Buske-Kirschbaum et al. (1997) | 1 | 0 | 1 | 1 | 2 | 0 | 1 | 1 | **7** |
| Camfferman et al. (2010) | 1 | 0 | 1 | 1 | 2 | 0 | 1 | 0 | **6** |
| Catal et al. (2016) | 1 | 0 | 0 | 1 | 0 | 0 | 1 | 0 | **3** |
| Horev et al. (2017) | 1 | 1 | 0 | 1 | 2 | 0 | 1 | 1 | **7** |
| Lee et al. (2016) | 1 | 1 | 1 | 1 | 2 | 1 | 1 | 1 | **9** |
| Sarkar et al. (2004) | 1 | 0 | 0 | 1 | 2 | 0 | 1 | 1 | **6** |
| Schmitt et al. (2009) | 1 | 0 | 0 | 1 | 2 | 1 | 1 | 1 | **7** |
| Shani-Adir et al. (2009) | 1 | 0 | 0 | 1 | 0 | 0 | 1 | 1 | **4** |
| Sule Afsar et al. (2010) | 0 | 0 | 0 | 1 | 2 | 0 | 1 | 1 | **5** |
| Urrutia-Pereira et al. (2017) | 1 | 1 | 0 | 1 | 2 | 0 | 1 | 1 | **7** |

1. **Quality Assessment of Cross-sectional Studies**

| **Study name** | **Selection** |  |  |  | **Comparability** | **Outcome** |  | **Total score** |
| --- | --- | --- | --- | --- | --- | --- | --- | --- |
|  | **Representativeness** | **Sample size** | **Non-respondents** | **Ascertainment of the exposure** | **Comparability of subjects in different outcome groups** | **Assessment** | **Statistical test** |  |
| Augustin et al. (2015) | 1 | 1 | 0 | 2 | 0 | 1 | 1 | **6** |
| Chang et al. (2013) | 1 | 1 | 1 | 2 | 0 | 1 | 1 | **7** |
| Chun et al. (2015) | 1 | 1 | 1 | 1 | 2 | 1 | 1 | **8** |
| Kuniyoshi et al. (2018) | 1 | 1 | 0 | 1 | 2 | 1 | 1 | **7** |
| Lee et al. (2017) | 1 | 1 | 1 | 1 | 2 | 1 | 1 | **8** |
| Lien et al. (2010) | 1 | 1 | 0 | 1 | 2 | 1 | 1 | **7** |
| Romanos et al. (2010) | 1 | 1 | 1 | 1 | 2 | 1 | 1 | **8** |
| Schmitt et al. (2018) | 1 | 1 | 0 | 1 | 0 | 1 | 1 | **5** |
| Silverberg et al. (2013) | 1 | 1 | 0 | 1 | 2 | 1 | 1 | **7** |
| Wang et al. (2017) | 0 | 1 | 0 | 2 | 2 | 1 | 1 | **7** |
| Yaghmaie et al. (2013) | 1 | 1 | 0 | 1 | 2 | 1 | 1 | **7** |
| Yang et al. (2018) | 1 | 1 | 0 | 1 | 2 | 1 | 1 | **7** |

**Appendix 2. Effect Sizes for Specific Mental Disorders**

**
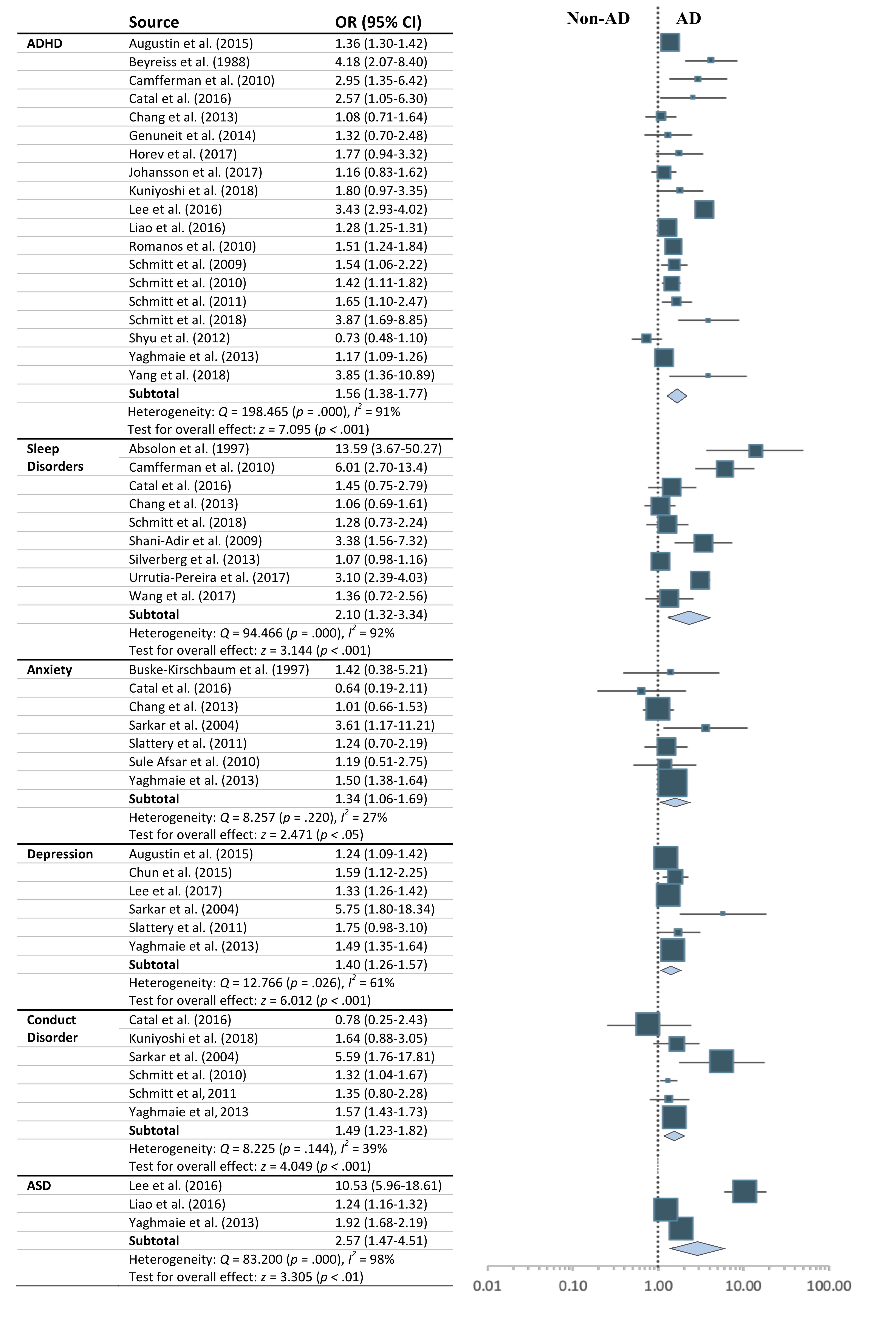
**

**Appendix 3. Public Bias**

1. **Total Mental Disorders**


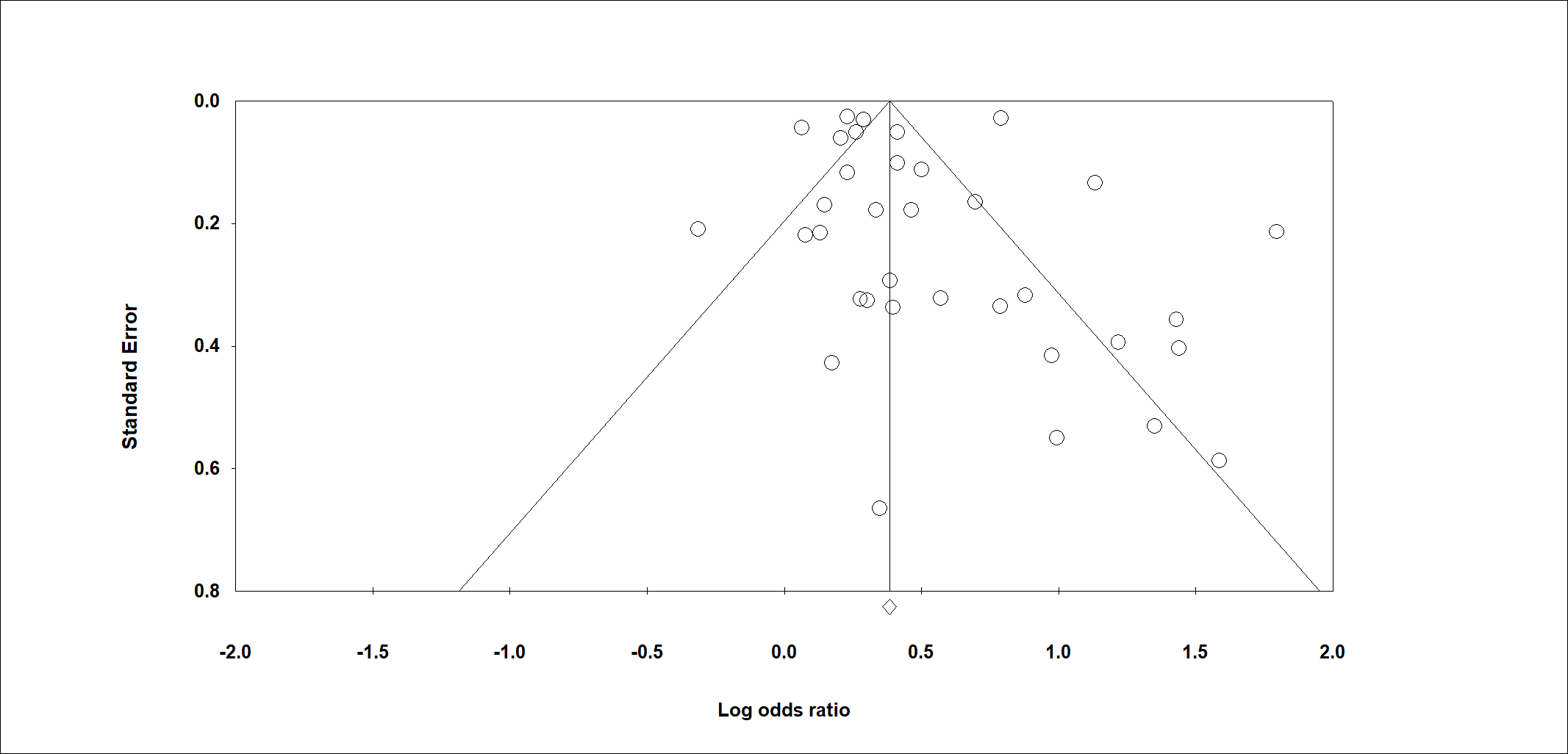


1. **ADHD**


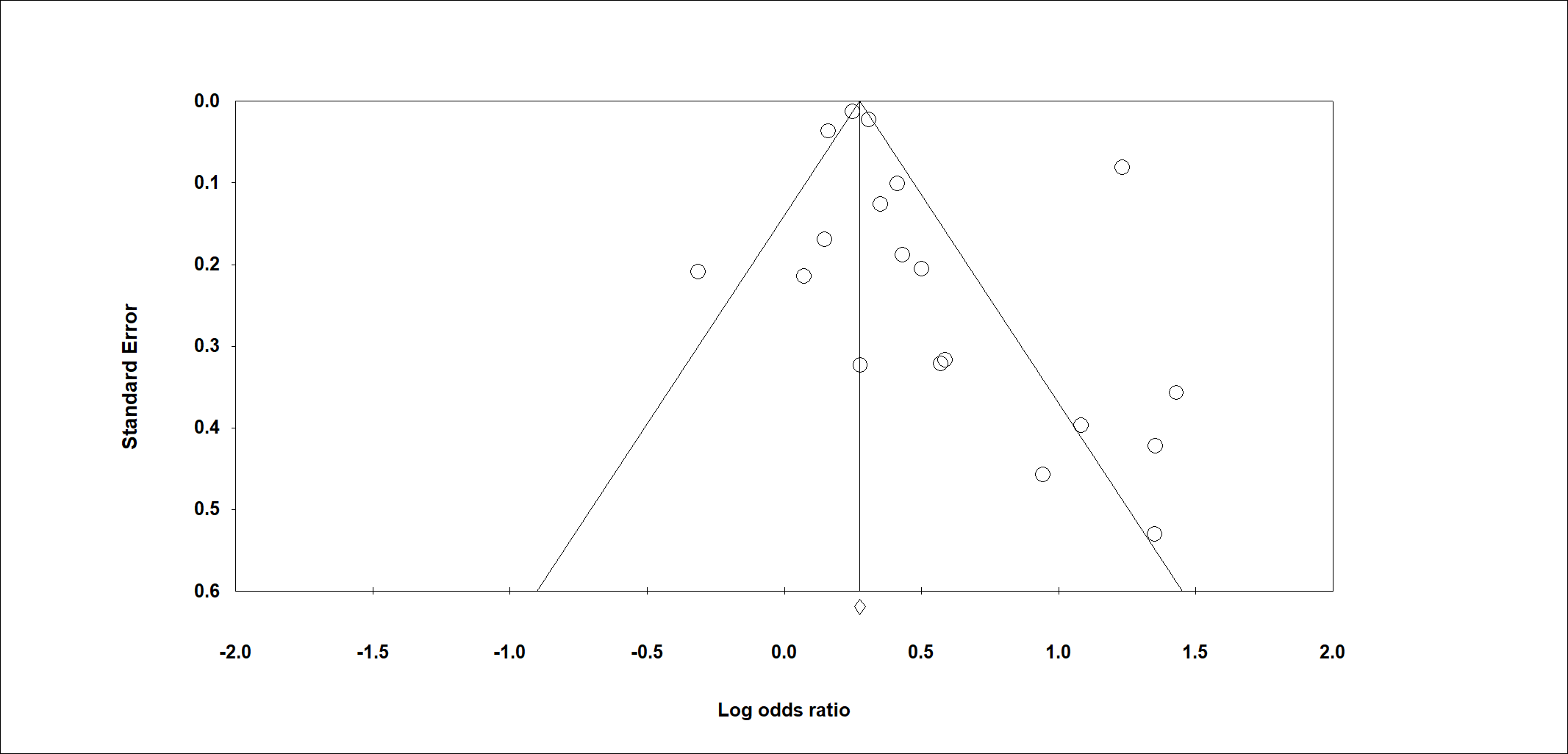


1. **Sleep Disorders**


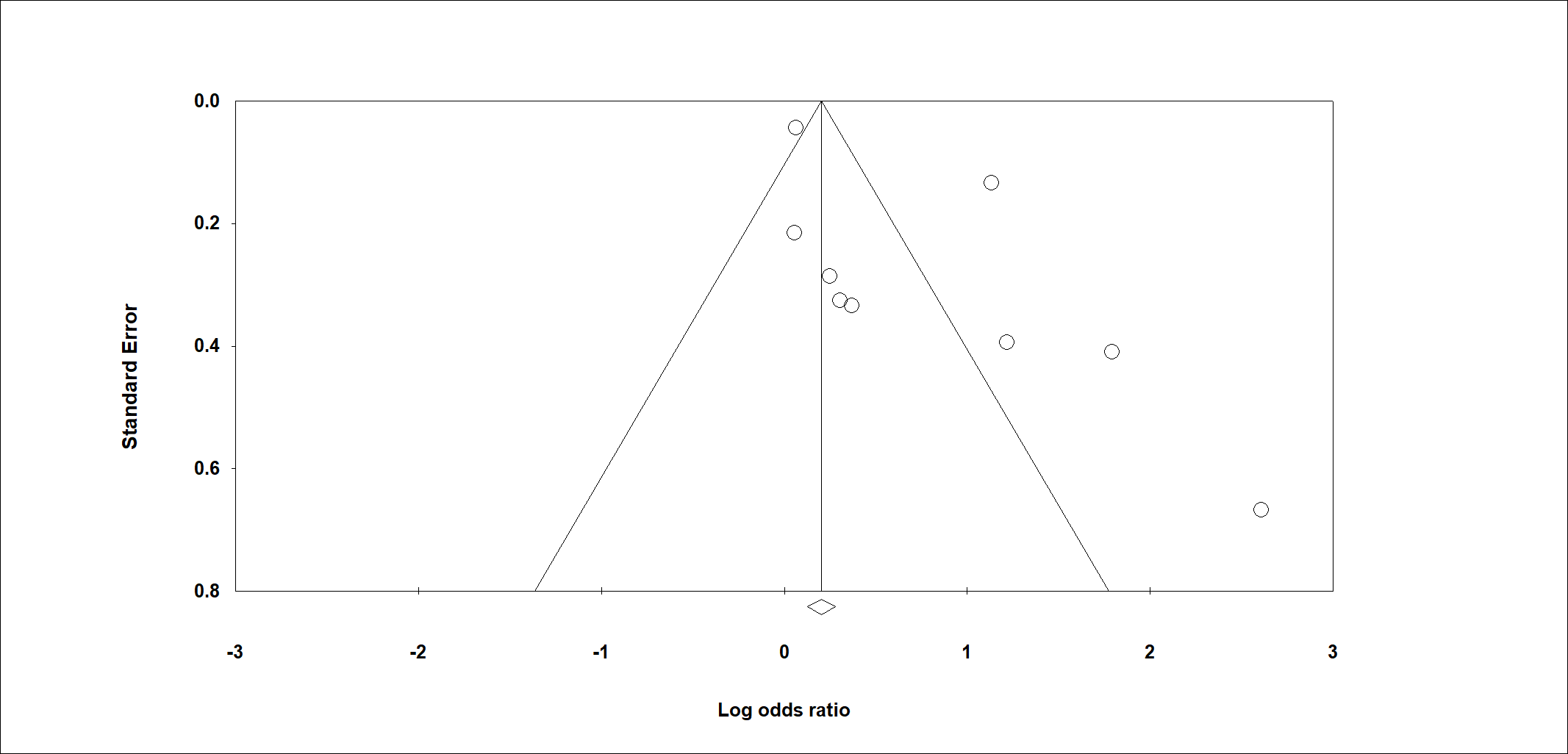


1. **Anxiety**


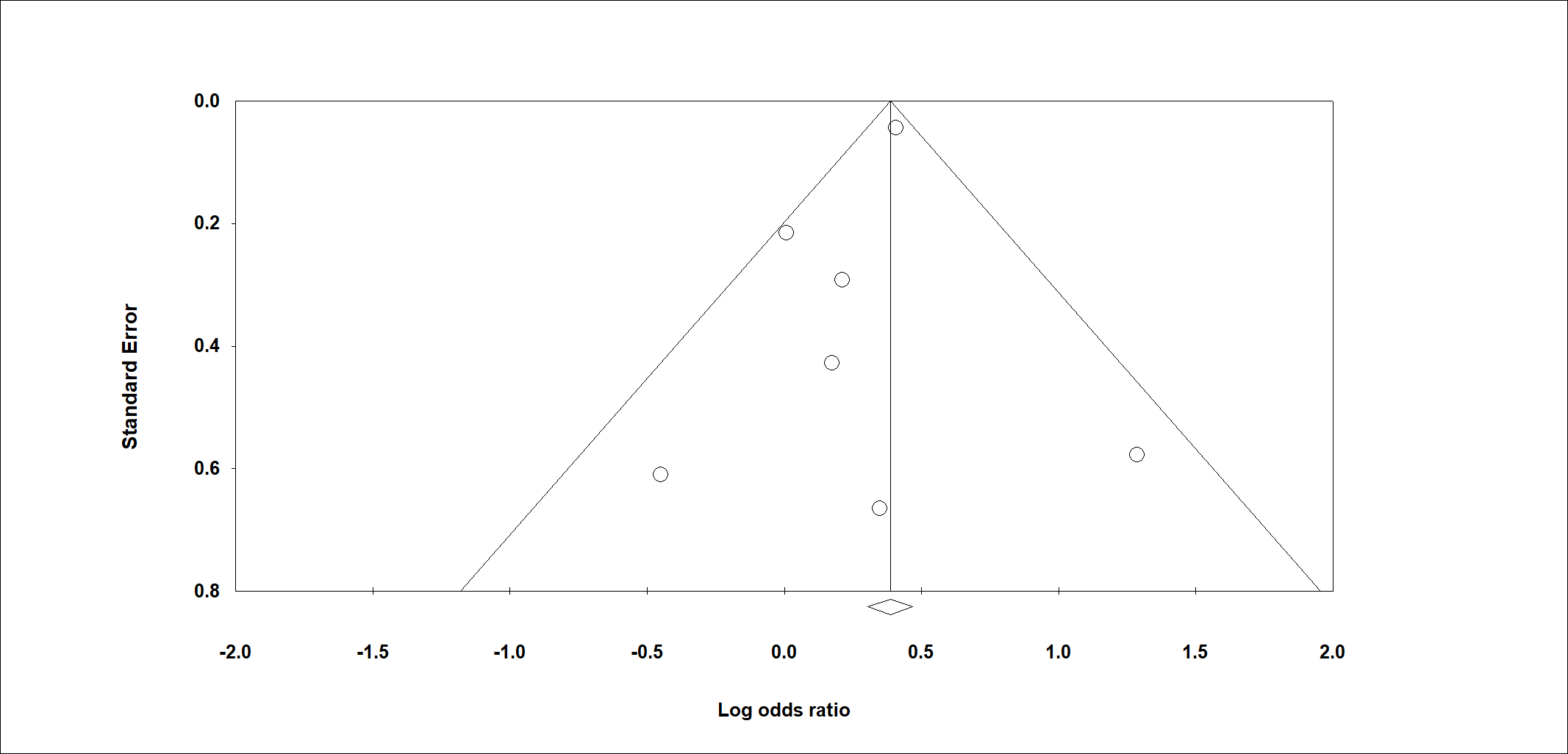


1. **Depression**


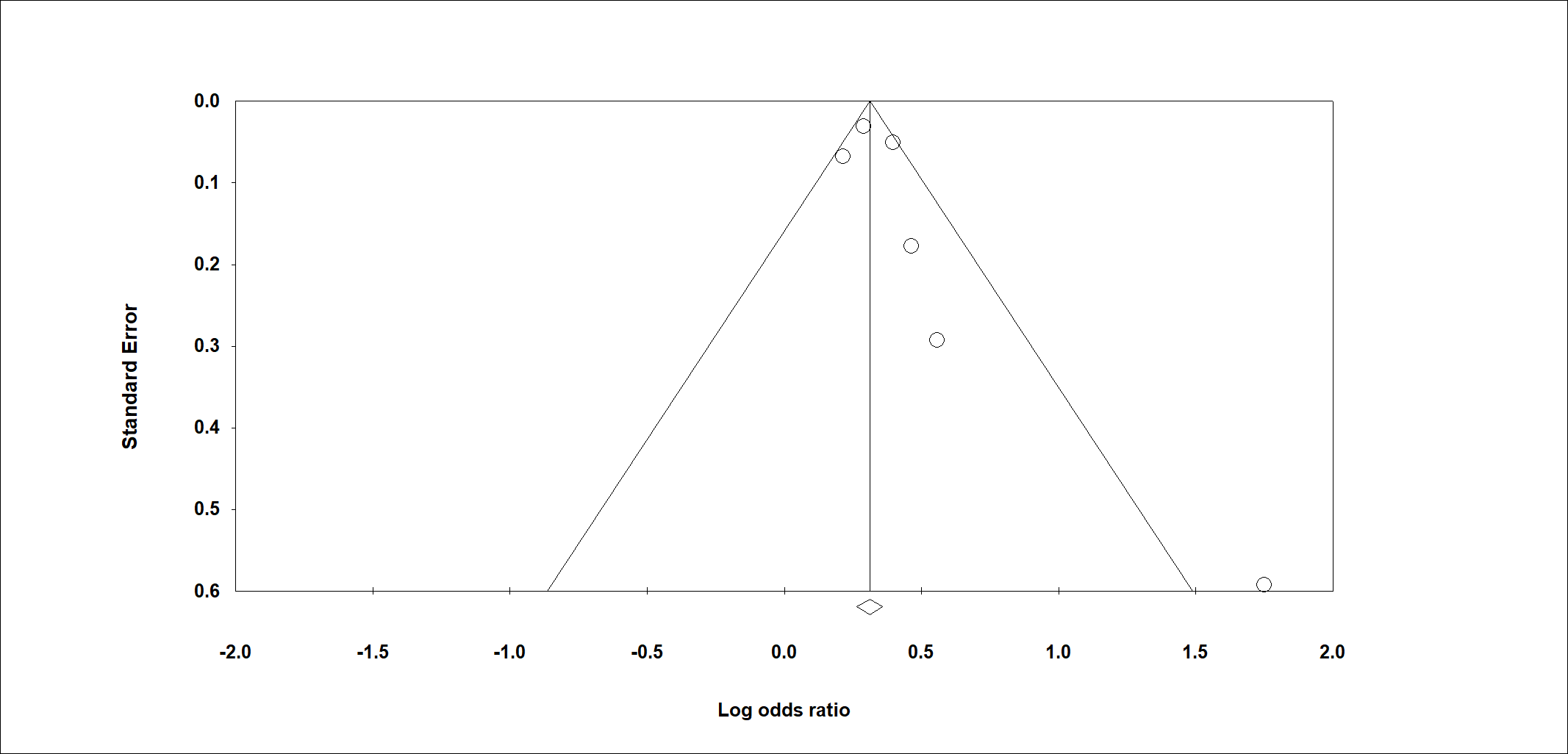


1. **Conduct Disorder**


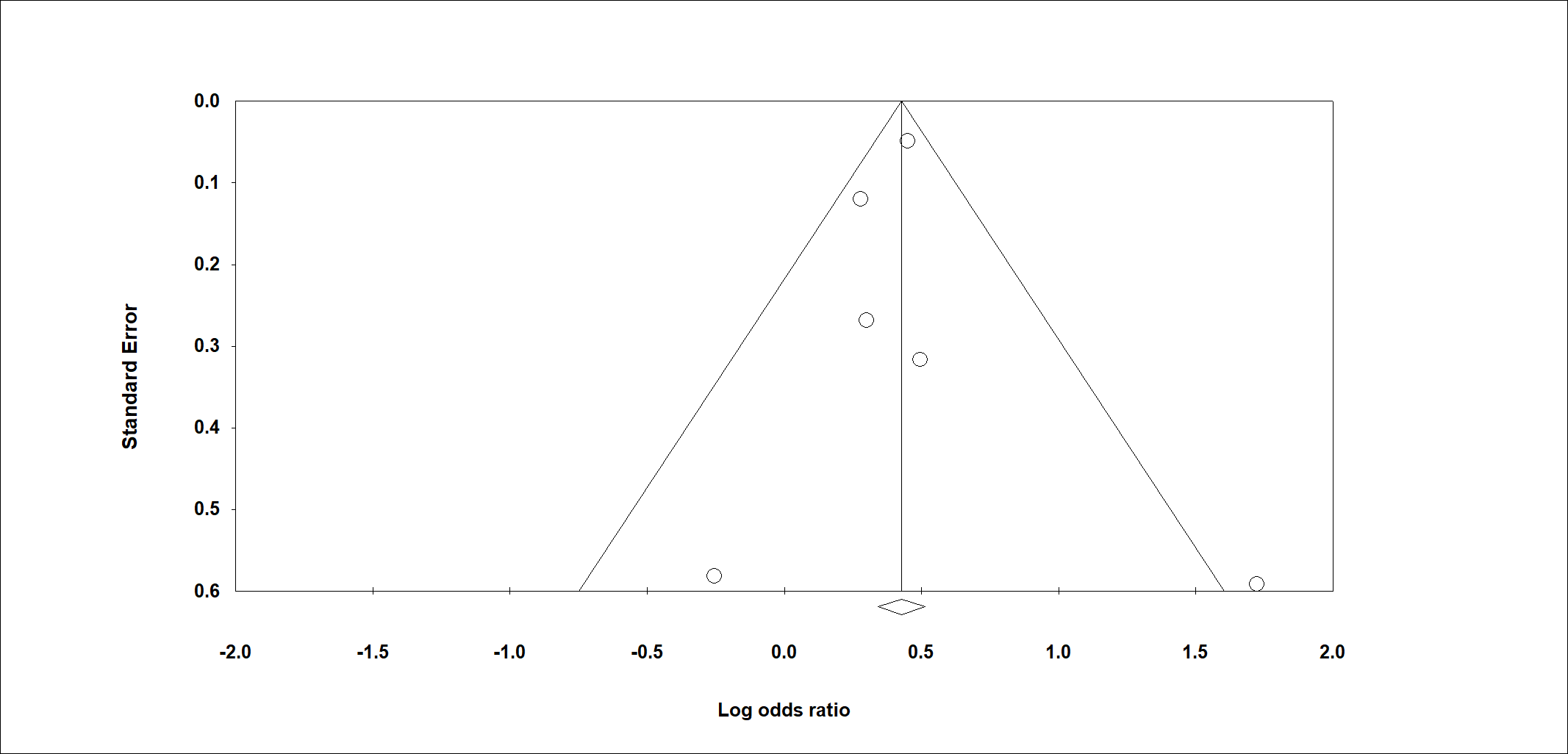


1. **ASD**


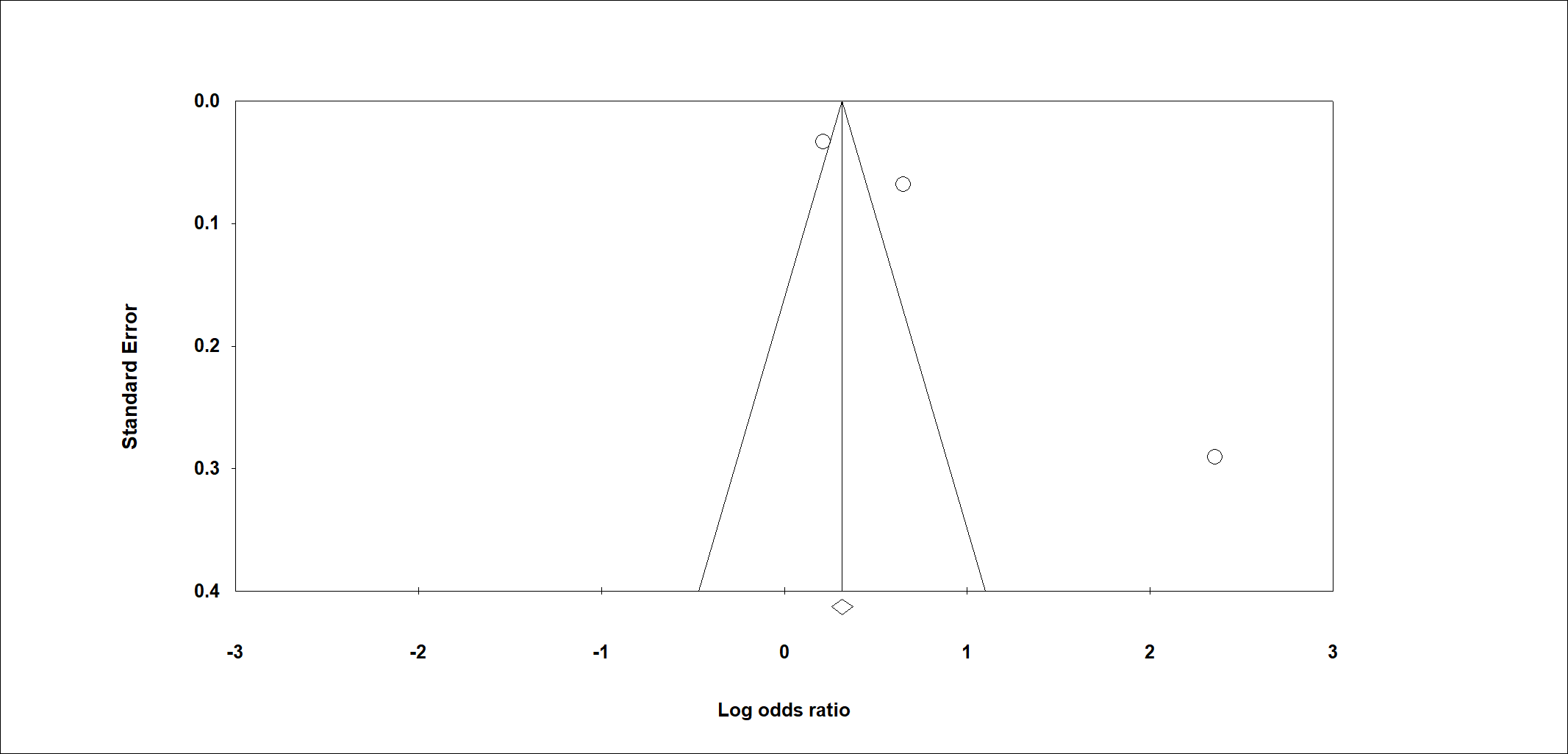

Supplement: Supplementary file 1 [file Table_1.DOCX]
